# Intra-Arterial thrombolysis after SUCCESSful angiographic recanalization in acute large-vessel occlusion stroke of the anterior circulation (IA-SUCCESS): a multicentre, randomised, clinical trial protocol

**DOI:** 10.1093/esj/aakag080

**Published:** 2026-07-13

**Authors:** Benjamin Gory, Sébastien Richard, Raoul Pop, Valérie Wolff, Aboubaker Cherifi, Martin Bretzner, Hilde Henon, Gaultier Marnat, Igor Sibon, Jean-Christophe Gentric, Serge Timsit, Fortunato Di Caterino, Guillaume Charbonnier, Jildaz Caroff, Christian Denier, Arturo Consoli, Bertrand Lapergue, Julien Allard, Gaspard Gerschenfeld, Victor Dumas, Matthias Lamy, Sebastian Johan Richter, Hélène Castagnet, Louis Veunac, Quentin Bourgeois-Beauvais, Jean-Philippe Desilles, Pierre Seners, Olivier Naggara, Anne-Christine Januel, Louis Fontaine, Grégoire Boulouis, Marco Pasi, Cyril Dargazanli, Caroline Arquizan, Olivier Heck, Katia Garambois, Pierre-Olivier Comby, Yannick Bejot, François Zhu, Liang Liao, Wassim Abou Loukoul, Anne-Laure Derelle, René Anxionnat, Marius Mkounga Kamga, Hamza Hachit, Francis Guillemin, Guillaume Turc, Gabriela Hossu

**Affiliations:** Department of Diagnostic and Therapeutic Neuroradiology, CHRU-Nancy, Nancy, France; Université de Lorraine, INSERM, IADI U1254, Nancy, France; Department of Neurology, Stroke Unit, Université, CHRU-Nancy, Nancy, France; INSERM U1116, CHRU-Nancy, Nancy, France; Department of Interventional Neuroradiology, Strasbourg University Hospitals, Strasbourg, France; Institut de Chirurgie Minime Invasive Guidée par l’Image, Strasbourg, France; INSERM UMR S1255, Etablissement Français du Sang, Strasbourg, France; Department of Stroke Unit, Strasbourg University Hospitals, Strasbourg, France; UR3072, CRBS, Strasbourg, France; CHRU-Nancy, INSERM, Université de Lorraine, CIC 1433, Innovation Technologique, Nancy, France; Department of Neuroradiology, University Hospital of Lille, Lille, France; Department of Neurology, University Hospital of Lille, Lille, France; Department of Diagnostic and Interventional Neuroradiology, University Hospital of Bordeaux, Bordeaux, France; Department of Neurology, University Hospital of Bordeaux, Bordeaux, France; Department of Neuroradiology, University Hospital of Brest, Brest, France; Department of Neurology, University Hospital of Brest, Brest, France; Department of Interventional Neuroradiology, University Hospital of Besançon, Besançon, France; Department of Interventional Neuroradiology, University Hospital of Besançon, Besançon, France; Department of Neurology, University Hospital of Besançon, Besançon, France; Department of Neuroradiology, University Hospital of Bicêtre, Paris, France; Department of Neurology, University Hospital of Bicêtre, Paris, France; Department of Neuroradiology, Foch Hospital, Versailles Saint-Quentin en Yvelines University, Suresnes, France; Department of Neurology, Foch Hospital, Versailles Saint-Quentin en Yvelines University, Suresnes, France; Department of Neuroradiology, Hôpital Pitié Salpêtrière, Paris, France; Department of Neurology, Hôpital Pitié Salpêtrière, Paris, France; Department of Radiology, University Hospital of Poitiers, Poitiers, France; Department of Neurology, University Hospital of Poitiers, Poitiers, France; Department of Radiology, Centre Hospitalier de Pau, Pau, France; Department of Neurology, Centre Hospitalier de Pau, Pau, France; Department of Radiology, Centre Hospitalier de Bayonne, Bayonne, France; Department of Neurology, Centre Hospitalier de Bayonne, Bayonne, France; Biological Resource Center and Department of Interventional Neuroradiology, Hôpital Fondation A. de Rothschild, Paris, France; Université Paris Cité, Paris, France; Department of Neurology, Hôpital Fondation A. de Rothschild, Paris, France; Department of Neuroradiology, Hôpital Saint Anne, Paris, France; Department of Neuroradiology, University Hospital of Toulouse, Toulouse, France; Department of Neurology, University Hospital of Toulouse, Toulouse, France; Diagnostic and Interventional Neuroradiology, CIC-IT 1415, Imaging Brain & Neuropsychiatry (iBraiN), UMR INSERM U1253, Tours University Hospital, Université de Tours, Tours, France; Department of Neurology, University Hospital of Tours, Tours, France; Department of Neuroradiology, Hôpital Gui de Chauliac, Montpellier University Medical Center, Montpellier, France; Department of Neurology, Hôpital Gui de Chauliac, Montpellier University Medical Center, Montpellier, France; Department of Neuroradiology, University Hospital of Grenoble, Grenoble, France; Department of Neurology, University Hospital of Grenoble, Grenoble, France; Department of Neuroradiology, University Hospital of Dijon, Dijon, France; Department of Neurology, University Hospital of Dijon, Dijon, France; Department of Diagnostic and Therapeutic Neuroradiology, CHRU-Nancy, Nancy, France; Université de Lorraine, INSERM, IADI U1254, Nancy, France; Department of Diagnostic and Therapeutic Neuroradiology, CHRU-Nancy, Nancy, France; Department of Diagnostic and Therapeutic Neuroradiology, CHRU-Nancy, Nancy, France; Department of Diagnostic and Therapeutic Neuroradiology, CHRU-Nancy, Nancy, France; Department of Diagnostic and Therapeutic Neuroradiology, CHRU-Nancy, Nancy, France; Université de Lorraine, INSERM, IADI U1254, Nancy, France; Department of Neuurology, CHR Metz-Thionville, Metz, France; CHRU-Nancy, INSERM, Université de Lorraine, CIC 1433, Epidémiologie Clinique, F-54000 Nancy, France; CHRU-Nancy, INSERM, Université de Lorraine, CIC 1433, Epidémiologie Clinique, F-54000 Nancy, France; Department of Neurology, GHU Paris Psychiatrie et Neurosciences, Université Paris Cité, Paris, France; Institute of Psychiatry and Neuroscience of Paris, INSERM U1266, Paris, France; Université de Lorraine, INSERM, IADI U1254, Nancy, France; CHRU-Nancy, INSERM, Université de Lorraine, CIC 1433, Innovation Technologique, Nancy, France

**Keywords:** endovascular treatment, intra-arterial thrombolysis, ischemic stroke, mechanical thrombectomy, randomized controlled trial, protocol

## Abstract

**Background:**

Intra-arterial thrombolysis (IAT) following successful mechanical thrombectomy (MT) in patients with anterior circulation large-vessel occlusion (LVO) improves cerebral tissue reperfusion and consequently clinical outcome, but has to be validated in non-Asian populations. We hypothesised that IAT with alteplase versus no IAT leads to a better clinical outcome in patients with anterior circulation LVO stroke who have successful angiographic recanalisation.

**Study design:**

Intra-Arterial thrombolysis after SUCCESSful angiographic recanalization in acute large-vessel occlusion stroke of the anterior circulation (IA-SUCCESS) trial is a phase 3 investigator-initiated, multicentre, randomised, open-label, blinded-endpoint (PROBE) clinical trial with a health economic evaluation conducted in France. Patients with acute ischaemic stroke due to anterior circulation LVO within 24 h of stroke onset and successful angiographic reperfusion (defined as extended Thrombolysis in Cerebral Infarction score 2b–3) after intravenous thrombolysis alone, MT alone or both will be randomised in two balanced parallel groups (1:1) to receive either IAT with alteplase (0.225 mg kg^–1^ and a maximum of 20 mg) injected in the ipsilateral internal carotid artery or no IAT. A total of 626 patients will be included.

**Study endpoints:**

The primary outcome is the functional outcome on the modified Rankin Scale at 90 (±15) days. Standard secondary clinical outcomes are assessed at 24 (±6) h, 5–7 days, 90 (±15) days and 12 (±1) months. Safety outcomes include mortality at 90 (±15) days and intracranial haemorrhage.

**Summary:**

The IA-SUCCESS trial will provide high-quality randomised data on the clinical efficacy and safety of intra-arterial alteplase following successful angiographic recanalisation in European patients with ischaemic stroke due to anterior circulation LVO intended for MT.

**Trial registration:**

ClinicalTrials.gov NCT06768138.

## Graphical Abstract

**Graphical Abstract:**
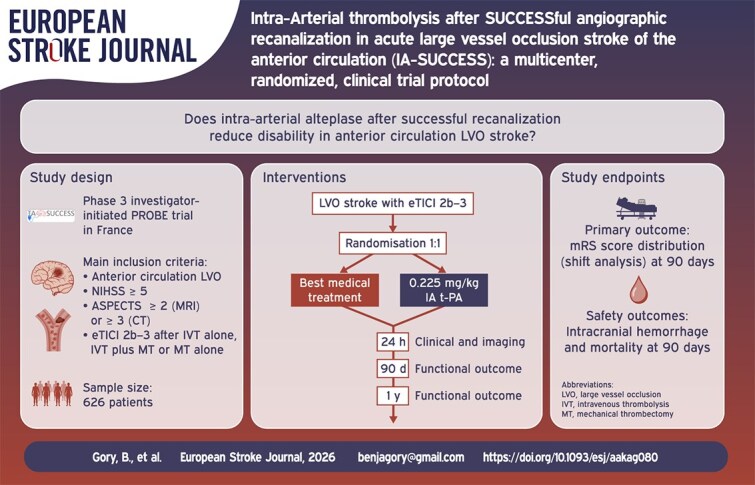

## Introduction and rationale

Mechanical thrombectomy (MT), with or without intravenous thrombolysis (IVT), is the standard of care for patients with ischaemic stroke due to anterior circulation large-vessel occlusions (LVO).^1^ Angiographic reperfusion grade is the most important modifiable predictor of patients’ outcome,^2^ and complete reperfusion (defined as expanded Thrombolysis in Cerebral Infarction [eTICI] grade 3) is achieved in only one-third of patients even with the last mechanical device generations.^3^ Adjunct intra-arterial thrombolysis (IAT) may be a promising therapeutic option allowing both distal arterial occlusion recanalisation (not accessible to mechanical devices) and improvement of downstream brain tissue reperfusion, according to the CHOICE pilot randomised trial.^4^^,^^5^ In addition, recent clinical evidence, even in the setting of complete reperfusion, has reported that patients can present failure of downstream microvascular reperfusion at the tissue level despite successful upstream macrovascular recanalisation (no-reflow) and this could be a major contributor to poor outcomes despite successful angiographic reperfusion (eTICI 2b–3).^6^ No-reflow is a well-known phenomenon in stroke animal models and after acute myocardial infarction in humans and is reported in one-third of recanalised LVO patients.^7^ In case of no-reflow, patients seem to have a similar final infarct volume and clinical outcomes as patients with unsuccessful recanalisation.^8^ Existing data on the added value of IAT after successful reperfusion for anterior circulation LVO is growing but remain conflicting and have been mainly conducted in Asian populations.^9–12^ In a recent meta-analysis including seven randomised controlled trials with over 2000 patients, the authors found that IAT was associated with a higher likelihood of excellent functional outcomes at 90 days without safety concerns.^13^ The effect was consistent across subgroups, especially in patients who received prior IVT and those with complete or near-complete angiographic reperfusion (eTICI 2c–3).^13^ However routine implementation of IAT after MT cannot yet be recommended due to the heterogeneity in study populations, treatment protocols (alteplase vs tenecteplase vs urokinase), the dose and the administration modality (local vs global). Therefore, large phase 3 multicentre randomised controlled trials to assess the efficacy and safety of IAT in stroke patients, particularly in non-Asian populations, are urgently needed.^14^ The Intra-Arterial thrombolysis after SUCCESSful angiographic recanalization in acute large-vessel occlusion stroke of the anterior circulation (IA-SUCCESS) trial aims to assess the safety and efficacy of IAT with alteplase in stroke patients with anterior LVO and angiographically confirmed successful recanalisation.

## Methods

### Study design

**Figure 1:**
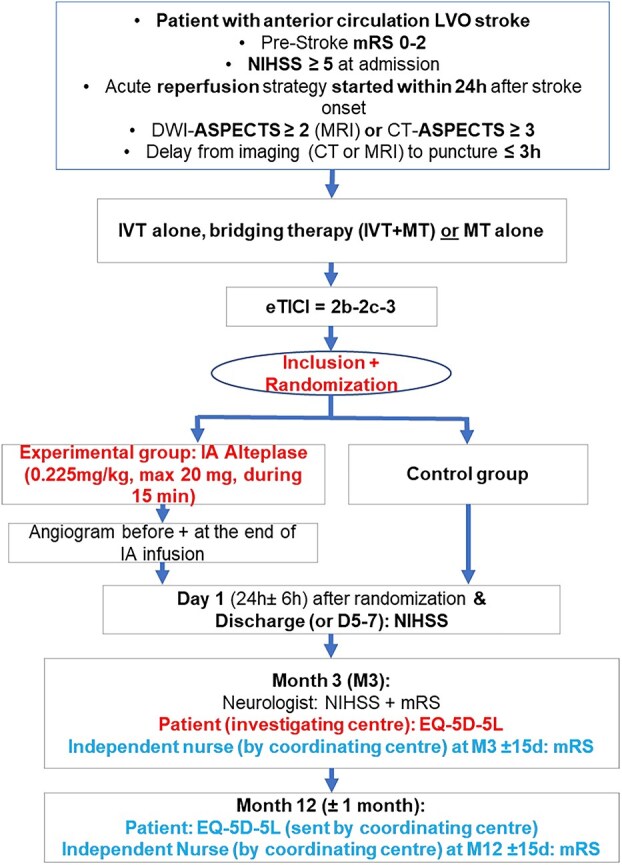
Study design of IA-SUCCESS trial. LVO: large-vessel occlusion; mRS: modified Rankin scale; NIHSS: National Institute of Health Stroke Scale; ASPECTS: Alberta Stroke Program Early CT Score; IVT: intravenous thrombolysis; MT: mechanical thrombectomy; eTICI: expanded thrombolysis in cerebral infarction; IA: intra-arterial; EQ-5D-5L: EuroQoL 5-dimensions 5-level questionnaire.

IA-SUCCESS is a phase 3, multicentre, prospective, randomised, open-label, blinded-endpoint design (PROBE), two-arms, clinical trial to compare the efficacy and safety of IAT with alteplase versus no IAT (standard of care) after angiographic confirmation of successful recanalisation in anterior LVO strokes intended for MT. Patients will be recruited from at least 20 large comprehensive stroke centres in France. Patient enrollment began in June 2025. The current protocol version in France is V1.1. The study is registered on ClinicalTrial.gov (NCT06768138) and is conducted in accordance with the declaration of Helsinki and Good Clinical Practice. Data collection will be carried out using an electronic case report form. The study design is summarised in Figure 1.

### Patient population

#### Inclusion criteria

- Age ≥18 years
- Pre-stroke modified Rankin Scale (mRS) score 0–2
- Acute ischaemic stroke with LVO defined as intracranial internal carotid artery or M1 or M2 of the middle cerebral artery proven on admission imaging (computed tomography [CT] or magnetic resonance imaging [MRI])
- National Institutes of Health Stroke Scale (NIHSS) ≥5 at admission
- Reperfusion therapy started within 24 h of onset according to international guidelines^15^^,^^16^
- Alberta Stroke Program Early Computed Tomography Score (ASPECTS) ≥2 on MRI (diffusion-weighted imaging [DWI]) or ≥3 on CT at admission
- Time from baseline imaging to arterial puncture within 3 h for transferred patients
- Successful reperfusion (eTICI 2b–2c–3) after IVT alone, thrombectomy alone or both confirmed by catheter angiogram
- Affiliation to or beneficiary of a social security plan

#### Exclusion criteria

- Contraindications for alteplase (platelets count <100,000, INR >1.7, direct oral anticoagulants taken within 48 h prior or biological confirmation of activity, effective treatment with heparin, etc)
- Haemorrhagic complications before or during MT
- Number of passes >5
- Intracranial haemorrhage
- Occlusion or high-grade stenosis of the internal carotid artery treated by stenting
- Any terminal illness such that patient would not be expected to survive more than 90 days
- Women of childbearing age without effective contraception
- Persons under involuntary psychiatric care, including compulsory admission or treatment without consent
- Pregnant, parturient or breastfeeding women; minors (non-emancipated); adults under legal protection (any form of guardianship or curatorship); persons deprived of liberty by judicial or administrative decision

### Randomisation

After emergent consent according to the French regulations, randomisation will be performed after angiographic confirmation of successful reperfusion (eTICI 2b–2c–3) in a 1:1 ratio using a centralised computer-generated minimisation algorithm to account for the following factors: centre, age (≤80 vs > 80 years), admission NIHSS (≤10 vs > 10), delay from onset to puncture (≤6 vs > 6 h), prior IVT (yes vs no, and alteplase vs tenecteplase) and MT (yes vs no), with a probability of 85% of allocating patients to the treatment group that minimises imbalance. Both patients and investigators will be aware of treatment allocation. The follow-up schedule is displayed in Table 1.

**Table 1:** Study visits.

	**Visit 1**	**Visit 2**	**Visit 3**	**Visit 4**	**Visit 5**
	**Baseline**	**24 ± 6 h**	**5–7 days or discharge** ^**a**^	**90 ± 15 days**	**12 ± 1 months**
**Medical history**	**S**				
**Clinical evaluation**	**S**	S	S	S	S
**Concomitant medications**	S	S	S	S	S
**NIHSS**	S	S	S	S	
**Neuroimaging (MRI or CT)^b^**	S	S			
**ASPECTS**	S	S			
**IVT (if performed)**	S				
**Thrombectomy (if performed)**	S				
**Angiogram (eTICI score)**	S				
**Screen for eligibility**	**R**				
**Informed consent**	**R**				
**Randomisation**	**R**				
**IAT (experimental group)**	**R**				
**mRS**	S^c^		S	S	
**Blinded mRS**				**R**	**R**
**TOAST classification**			**S**		
**Adverse events**	**R**	**R**	**R**	**R**	**R**
**EuroQol EQ-5D-5L**				**R**	**R**

R: research; S: standard of care; NIHSS: National Institute of Health Stroke Scale; ASPECTS: Alberta Stroke Program Early CT Score; IVT: intravenous thrombolysis; eTICI: expanded Thrombolysis In Cerebral Infarction; mRS: modified Rankin Scale; IAT: intra-arterial thrombolysis; EQ-5D-5L: EuroQoL 5-Dimensions 5-Level Questionnaire; TOAST: Trial of ORG 10172 in Acute Stroke Treatment.

^a^Visit 3 will take place on day 7 ± 1 or the day of discharge, whichever comes first.

^b^Non-contrast CT at visit 2 is sufficient.

^c^Pre-stroke mRS.

### Treatment or intervention

Patients eligible for enrollment are randomised by investigators (neuroradiologists or neurologists) in a 1:1 ratio to IAT with alteplase (experimental group) at a dose of 0.225 mg kg^–1^ (maximum dose of 20 mg) or no IAT (control group) after angiographic confirmation of successful reperfusion defined as eTICI 2b–2c–3. The overall intra-arterial alteplase dose is injected through the guiding catheter placed in the ipsilateral internal carotid artery over a period of 15 min. A final angiographic run is performed at the end of IA infusion in order to assess the final intracranial reperfusion status. In the control group, no placebo treatment is administered.

### Outcomes

The primary outcome is the degree of disability at 90 (±15) days on the mRS score distribution (shift analysis). The mRS score is a disability scale ranging from 0 (no symptoms at all) to 6 (death).^15^ This primary outcome will be assessed by independent, centralised, trained and certified nurse blinded to randomisation group and patients’ characteristics using the Rankin Focused Assessment.^16^ The mRS score will be assessed by telephone using a structured questionnaire for all patients from the nurse of the investigator centre. The pre-defined secondary outcomes included: (1) excellent functional outcome (mRS 0–1) at 90 (±15) days and 12 (±1) months; (2) favourable functional outcome (mRS 0–2) at 90 (±15) days and 12 (±1) months; (3) early neurological improvement defined as reduction in NIHSS by at least 8 points or NIHSS score of 0–1 at 24 (±6) h; (4) final eTICI; (5) infarct growth at 24 (±6) h; (6) quality of life as assessed by the EuroQoL 5-Dimensions 5-Level Questionnaire at 90 (±15) days and 12 (±1) months; (7) cost effectiveness analysis defined as incremental cost-effectiveness and cost-utility ratios at 1 year of a strategy based on adding IAT after successful angiographic reperfusion with a strategy of not adding IAT; and (8) total cost defined as total cost of each treatment strategy and net impact to the National Health Insurance System (difference in cost).

The safety outcomes are: (1) asymptomatic and symptomatic intracranial haemorrhage (sICH) at 24 (±6) h on imaging follow-up, validated by an imaging core laboratory, according to the Heidelberg bleeding classification,^17^ and as assessed centrally by an independent clinical events committee (distinct from the data safety monitoring board [DSMB] and composed of three vascular neurologists); (2) severe systemic bleedings before discharge; (3) decompressive craniectomy before discharge; and (4) all-cause mortality before discharge and at 90 (±15) days. These safety outcomes will be evaluated throughout the study period and documented in safety annual reports transmitted to competent authorities and ethical committees.

### Data safety monitoring board

The study will be monitored by a DSMB that consists of independent clinicians including one vascular neurologist, one neuro-interventionist and one independent statistician. The DSMB will provide safety oversight and make recommendations to the sponsor regarding continuation, modification or termination of the study based upon review of the comparative rates of safety events including sICH according to the Heidelberg bleeding classification, parenchymal haematoma type 2 on 24-h imaging, any systemic bleeding requiring transfusion or surgery, and all-cause mortality within 90 (±15) days. The DSMB meetings will be organised by call conference by the sponsor before the start of the study and after every 10 serious adverse events of interest for the two first meetings, then after every 20 serious adverse events of interest until the end of the study according to the DSMB advice. The DSMB will also receive the results of interim analyses. Additional extraordinary meetings will be set if necessary.

### Sample size estimation

The sample size calculation is based on the following hypotheses and uses a two-sided two sample comparison of ordinal outcomes for an ordinal shift analysis: (1) a common odds ratio (cOR) for better functional outcome (improvement in the 90-day mRS score of at least one point) of 1.5, which is consistent with the treatment effect observed in the CHOICE trial (cOR of 1.54);^18^ and (2) the following 90-day mRS distribution for the control group based on unpublished data (*n* = 3767 patients matching the inclusion criteria of IA-SUCCESS) from the ongoing observational Endovascular Treatment in Ischemic Stroke (ETIS, NCT03776877) registry in France between January 2015 and December 2023 (mRS score 0 of 14.4%, mRS score 1 of 18.9%, mRS score 2 of 15.3%, mRS score 3 of 15.3%, mRS score 4 of 11.4%, and mRS score 5–6 of 24.6%). Based on these assumptions, we calculated that 298 evaluable patients per treatment group would provide a power of at least 80% to detect a difference in the distribution of 90-day mRS scores with a two-sided significance level of 0.05, using Tang’s exact Wilcoxon–Mann–Whitney power formula for ordinal outcomes. To account for an anticipated attrition rate of approximately 5% (including loss to follow-up or missing primary outcome data), the total sample size was increased to 626 patients, corresponding to 313 patients per group. A sensitivity analysis was performed by applying modest variations to the assumed control-group mRS distribution, consisting of a 5 percentage-point shift between the most favourable (mRS 0–1) and least favourable (mRS 5–6) outcome categories. The resulting required sample sizes ranged from 592 to 598 patients, compared with 596 patients for the reference ETIS-based distribution, supporting the robustness of the sample size estimate.

### Statistical analysis

All participants who are randomly assigned will be included in the full analysis set (FAS) following the intention-to-treat (ITT) principle. The FAS is the primary efficacy evaluation population. All randomised participants will be included in the FAS. Consistent with the ICH E9, the FAS is defined as the analysis set that is as complete as possible and as close as possible to the ITT principle of including all randomised participants. The FAS will constitute the primary efficacy evaluation population for this trial. The primary analysis will be performed on participants with available 90-day mRS data. To address the primary objective, the distribution of the 90-day mRS scores will be compared between randomisation groups (ITT analysis), with scores of 5 or 6 collapsed into a single group. For this purpose, the shift in 90-day mRS toward a better functional outcome (ie, improvement of at least one mRS point compared with the control group) will be estimated using an ordinal logistic model regression for minimisation variables (except centre), provided that the proportional odds assumption is satisfied. Centre was intentionally not included in the primary adjusted model because of the relatively large number of participating centres compared with the planned sample size, in order to avoid convergence problems and overparameterisation. A sensitivity analyses including centre as a random effect may be performed to assess the robustness of the results. The proportional odds assumption will be assessed using the Brant test, with a two-sided *P* value <.05 considered indicative of violation of the assumption. A cOR with 95% confidence interval (CI) will then be derived. If the proportional odds assumption is not satisfied, the primary outcome will be analysed using a partial proportional odds model. If the proportional odds assumption is violated, treatment effect estimates will be reported using Wilcoxon–Mann–Whitney generalised odds ratios (WMW GenOR). This approach is consistent with current practice in recent stroke trials using ordinal mRS outcomes. The per-protocol (PP) population will include randomised patients analysed according to their assigned group who received the allocated strategy and had no major protocol violations. Patients will be excluded from the PP population in case of: (1) failure to receive IAT in the experimental group, (2) major protocol violations, including violation of entry criteria or (3) unavailable measurement of the primary outcome. In case of missing data for the primary outcome (90-day mRS score), analyses will be performed on the available data without further specification under the assumption that data are missing at random. No imputation will be performed for the primary analysis. Sensitivity analyses may be conducted, if appropriate, to assess the robustness of the results to missing outcome data. Statistical analyses will be performed by independent statisticians unaware of the group allocation. One interim analysis including the analysis of the primary outcome will be conducted after inclusion of 50% of the planned sample size. The significance threshold for this interim analysis is set at .001 according to the Haybittle–Peto method, which uses a very conservative interim significance level in order to preserve an overall two-sided type I error rate of 5% for the trial. A cost-utility analysis will be conducted by using quality of life scores collected at 12 months of follow-up, to estimate the cost per one quality-adjusted life year gained. In addition to cost-effectiveness and cost-utility analysis, a budget impact analysis will be performed to estimate the total cost of adopting the IAT of treatment at the national level and the expected number of disability cases averted. All analyses and substudies planned within the statistical analysis plan have been described in the manuscript. The following prespecified subgroup analyses will be considered exploratory and hypothesis-generating. No adjustment for multiple comparisons will be performed, and subgroup findings will therefore be interpreted with caution. The following prespecified subgroups will be explored in sensitivity analyses:

1. Sex: males vs females
2. Use of IVT at admission: yes vs no, alteplase vs tenecteplase
3. Age: ≤80 vs >80 years
4. NIHSS at admission: 5–10 vs > 10
5. DWI-ASPECTS at admission: ≤5 vs > 5
6. Time from onset or last well known to puncture: ≤6 vs > 6 h
7. Time from IVT to IAT: cut-off median delay
8. Final eTICI: 2b vs 2c–3 and 2b vs 2c vs 3
9. Imaging at admission: MRI vs CT
10. Mothership vs dirp and ship

## Discussion

In the recent endovascular treatment era, IAT has gained attention as adjunct therapy following successful endovascular reperfusion or in case of non-accessible distal occlusion. Currently, many trials are investigating the efficacy and safety of IAT after MT in anterior circulation LVO.^4^^,^^9–12^^,^^19^^,^^20^ Some of them test IAT with tenecteplase (with different regimens),^10^^,^^11^^,^^20^ others with alteplase^4^^,^^12^^,^^19^ or urokinase,^8^ with conflicting results. In a recent meta-analysis including 1083 patients treated with IAT and 1048 patients without IAT, the authors found that IAT was associated with higher likelihood of excellent functional outcome (relative risk: 1.23; 95% CI: 1.11–1.36; I^2^ = 0%) and reduced disability at 3 months (cOR: 1.10; 95% CI: 1.03–1.18; I^2^ = 0%).^13^ Similar rates of 3-month good functional outcome, 3-month mortality, and asymptomatic and symptomatic ICH were observed.^13^ However, significant heterogeneity in the design, patient population and procedural metrics across studies were noted which may contribute to the variance of treatment effect reported across trials. The predominance of Asian populations also limited international generalisation. To date, including the IA-SUCCESS trial, four clinical trials are evaluating IAT with alteplase in anterior circulation LVO stroke patients which are finished or still ongoing (Table 2). The PEARL trial (NCT05856851) included 324 patients in China and found a higher rate of 90-day mRS score 0–1 (44.8% vs 30.2%, adjusted risk ratio 1.45; 95% CI 1.08–1.96) after IAT (0.225 mg kg^–1^, maximum dose 20 mg).^12^ In non-Asian populations, there are only two phase 3 trials: CHOICE 2 and IA-SUCCESS. The CHOICE 2 trial included 440 patients in Spain and found that 57.5% of patients (123/214) in the MT plus IAT alteplase (0.225 mg kg^–1^, maximum dose 20 mg) group had a 90-day mRS of 0 or 1 compared with 42.5% of patients (93/219) in the MT alone group (adjusted risk difference, 15.0%; 95% CI: 5.7%–24.3%; *P* = .002) without a significant increase in sICH.^19^ In addition, mortality at 90 days was higher in the MT plus IAT alteplase group compared with the MT alone group (12.1% vs 6.4%; adjusted risk difference, 5.9%; 95% CI: 0.5%–11.3%; *P* = .03).^19^ Any evident explication was found and IAT is to date the standard of care for all patients.^21^ In IA-SUCCESS, a total of 626 patients randomised will provide 80% statistical power to detect a cOR for better functional outcome (improvement in the 90-day mRS score of at least one point) of 1.5, as observed in the CHOICE trial.^4^ We decided to use a shift analysis for the primary endpoint due to the broader eligibility criteria, such as large core and recanalisation with IVT alone, compared with other trials. Compared with others trials, patients with large core (ASPECTS 2–5) on baseline imaging and with early recanalisation after IVT alone could be included in IA-SUCCESS trial. IA-SUCCESS is currently ongoing in 20 sites in France. As of 21 May 2026, a total of 285 patients have been randomised.

**Table 2:** Major methodological differences between the four trials investigating IAT with alteplase in anterior circulation LVO.

	**IA-SUCCESS**	**CHOICE**	**CHOICE 2**	**PEARL**
**ClinicalTrials**	NCT06768138	NCT03876119	NCT05797792	NCT05856851
**Design**	3	2b	3	3
**Hypothesis**	80% power to detect a difference with a cOR of 1.50 for an improvement in the 90-day mRS score of at least one point	80% power to detect an absolute improvement of 21% in the primary outcome (90-day mRS 0–1), assuming a proportion of 40% in the control group	80% power to detect a14% absolute benefit in the primary outcome (90-day mRS 0–1), assuming a rate of 40% in the control group	80% power to detect an absolute improvement of 16% in the primary outcome (90-day mRS 0–1), assuming a proportion of 40.4% in the control group
**Sample size**	626	121	440	324
**Country**	France	Spain	Spain	China
**Study population**	Age ≥18 years	Age ≥18 years	Age ≥18 years	Age ≥18 years
	Pre-stroke mRS 0–2	Pre-stroke mRS 0–1	Pre-stroke mRS 0–1	Pre-stroke mRS 0–1
	0–24 h of LKW	0–24 h of LKW	0–24 h of LKW	0–24 h of LKW
	eTICI 2b–3	mTICI 2b–3	mTICI 2b–3	eTICI 2b–3
**NIHSS**	NIHSS ≥5	NIHSS ≤ 25	NIHSS ≤ 25	NIHSS 6–25
**Core size**	ASPECTS 2-10 (DWI-MRI) or 3-10 (CT)	ASPECTS 6-10	ASPECTS 6-10	ASPECTS 6-10 (CT or MRI)
**Occlusion site**	ICA or MCA	ICA, ACA or MCA	ICA, ACA or MCA	ICA or MCA
**Reperfusion strategy**	IVT alone, IVT + MT or MT alone	IVT + MT or MT alone	IVT + MT or MT alone	IVT + MT or MT alone
**Alteplase dose**	0.225 mg kg^–1^ during 15 min	0.225 mg kg^–1^ during 15 min	0.225 mg kg^–1^ during 15 min	0.225 mg kg^–1^ during 15 min
	Maximum dose 20 mg	Maximum dose 22.5 mg	Maximum dose 20 mg	Maximum dose 20 mg
**Primary endpoint**	90-day mRS (shift analysis)	90-day mRS 0–1	90-day mRS 0–1	90-day mRS 0–1

ASPECTS, Alberta Stroke Program Early Computed Tomography Score; cOR, common odds ratio; CT, computed tomography; DWI, diffusion-weighted imaging; eTICI, expanded Thrombolysis in Cerebral Infarction; IAT, intra-arterial thrombolysis; IVT, intravenous thrombolysis; LVO, large-vessel occlusion; MRI, magnetic resonance imaging; MT, mechanical thrombectomy; NIHSS, National Institutes of Health Stroke Scale; mRS, modified Rankin scale; LKW, last known well; mTICI, modified Thrombolysis in Cerebral Infarction; ICA, internal carotid artery; ACA, anterior cerebral artery; MCA, middle cerebral artery.

## Summary and conclusions

IA-SUCCESS is a large phase 3 multicentre randomised clinical trial with a PROBE design. We hope to demonstrate superiority of IAT with alteplase after angiographic recanalisation in European stroke patients with anterior circulation LVO intended for MT, which would impact the current guidelines regarding treatment recommendations.
